# Use of electronic nicotine delivery systems and other tobacco products among USA adults, 2014: results from a national survey

**DOI:** 10.1007/s00038-015-0761-0

**Published:** 2015-11-12

**Authors:** Scott R. Weaver, Ban A. Majeed, Terry F. Pechacek, Amy L. Nyman, Kyle R. Gregory, Michael P. Eriksen

**Affiliations:** Division of Epidemiology and Biostatistics, School of Public Health, Georgia State University, Atlanta, GA USA; Tobacco Center of Regulatory Science (TCORS), School of Public Health, Georgia State University, Atlanta, GA USA; Division of Health Management and Policy, School of Public Health, Georgia State University, Atlanta, GA USA

**Keywords:** Electronic nicotine delivery systems, Electronic cigarette, Tobacco, Prevalence, Awareness, Regulatory science

## Abstract

**Objectives:**

This study assessed the awareness and use of traditional and novel tobacco products and dual use of cigarettes with electronic nicotine delivery systems (ENDS) among USA adults.

**Methods:**

Data were obtained from the 2014 Tobacco Products and Risk Perceptions Survey of a probability sample of 5717 USA adults conducted June–November, 2014.

**Results:**

Use of ENDS varied by demography and by cigarette and other tobacco use. Adults aged 25–34, non-heterosexual adults, and those reporting poorer health reported higher rates of current ENDS use. Current cigarette smokers had much greater odds of ENDS ever use than never smokers, with one-half of all cigarette smokers having used ENDS and 20.7 % currently using them. However, 22.0 % of current ENDS users were former cigarette smokers, and 10.0 % were never cigarette smokers.

**Conclusions:**

Patterns of ENDS use are evolving rapidly and merit continued surveillance. Nearly 10 % of adult ENDS usage is among never smokers. The public health challenge is how to enhance the potential that ENDS can replace combusted tobacco products without expanding nicotine use among youth, long-term ex-smokers, and other vulnerable populations.

**Electronic supplementary material:**

The online version of this article (doi:10.1007/s00038-015-0761-0) contains supplementary material, which is available to authorized users.

## Introduction

The USA appears to be entering a period of dramatic transformation of the types of tobacco products commonly used in the USA, potentially as dramatic as when the “modern” cigarette replaced the cigar and chewing tobacco 100 years ago, and when the filtered and “low-tar” cigarette transformed the market 40–60 years ago (US Department of Health and Human Services 2014). The latter transformation was driven by the increased knowledge of the health risks of cigarettes (e.g., 1964 Surgeon General Report) but produced a shift in product that did not reduce population harm and increased individual risk for some tobacco-related diseases such as adenocarcinoma of the lung (US Department of Health and Human Services 2014). As societal knowledge about the harm caused by traditional cigarettes has continued to increase, the number and type of tobacco and nicotine products available to consumers have increased dramatically in recent years. The current array of novel and alternative products includes non-combustible, smokeless, spit-less tobacco products such as snus and dissolvables, which differ in form from conventional chewing tobacco (Bahreinifar et al. 2013; Seidenberg et al. 2012); little cigars and cigarillos (LCCs), which differ from their larger, conventional cigar counterparts; water pipes or hookahs; and an increasing variety of electronic vapor products (ENDS), known most commonly as e-cigarettes (Agaku and Alpert 2015; Agaku et al. 2014; McMillen et al. 2014). ENDS vary widely in appearance and functionality, but all are battery-powered and can deliver combinations of nicotine and other additives in an aerosol (Knorst et al. 2014; Orellana-Barrios et al. 2015).

Awareness of many of these novel and alternative products appears to be significant and rising steadily among adults in the USA (Choi and Forster 2013; King et al. 2015). Studies conducted on the prevalence of use for these products indicate some volatility in popularity of the newer smokeless products (Biener et al. 2014; Zhu et al. 2013) but substantial and rising prevalence rates for little cigars and cigarillos among certain subgroups (Cohn et al. 2015; Corey et al. 2014; Messer et al. 2015; Richardson et al. 2013), hookahs (Brockman et al. 2012; Villanti et al. 2015), and, especially, for ENDS (Agaku et al. 2014; Carroll Chapman and Wu 2014; King et al. 2015; McMillen et al. 2014; Pepper and Brewer 2014).

High rates of smoking-attributable disease and death, and the associated health and societal costs, have been predicted to persist for decades into the 21st century (US Department of Health and Human Services 2014). These disturbing predictions have prompted some to encourage this current pattern of tobacco product transformation, hoping that the novel products could develop into a much lower risk “disruptive technology” that replaces the most lethal forms of nicotine delivery: the cigarette and the other inhalable combusted products (Abrams 2014; Cobb and Abrams 2014; Fagerstrom and Bridgman 2014; West and Brown 2014). However, others have raised cautions about how these new products could produce harms to population health (Chapman 2015; Chen and Husten 2014; England et al. 2015; Grana et al. 2014; Schraufnagel et al. 2014).

As of October, 2015, the Food and Drug Administration lacks the authority to regulate several novel tobacco products, notably ENDS marketed for non-therapeutic purposes (Chen and Husten 2014). Despite meeting the definition of a “tobacco product” under the Family Smoking Prevention and Tobacco Control Act (FSPTCA), the act only granted the FDA immediate authority over cigarettes, smokeless tobacco, and roll-your-own tobacco (Chen and Husten 2014). However, the FSPTCA allows the FDA to promulgate regulations extending regulatory authority over all other products meeting the definition of tobacco product (Lindblom 2015). The FDA needs current information on rates and trends of use of these products to guide and support its rule making and regulatory actions (or lack thereof).

In summary, the public health challenge is how to balance the public health messaging about these alternative and novel tobacco products and to enhance the potential that they can become a “disruptive technology” replacing the combusted tobacco products without expanding the patterns of nicotine use among youth and young adults, long-term ex-smokers, and other vulnerable populations. Information on how these alternative and novel tobacco products are being used is critically needed as the marketing and use of these products are rapidly increasing (Emery et al. 2014; Pepper et al. 2015). To address these needs, data from the 2014 Tobacco Products and Risk Perceptions Survey are presented here assessing the awareness and use of ENDS and other novel or alternative tobacco products among USA adults, demographic predictors of use, and dual use of combusted tobacco products and ENDS. We are not aware of any other published prevalence estimates of USA adult ENDS use from 2014. Together, these results will help inform public health and regulatory decisions about the rapidly evolving ENDS products.

## Methods

### Data source

We obtained data for this study from the 2014 Tobacco Products and Risk Perceptions Survey conducted by the Georgia State University Tobacco Center of Regulatory Science (TCORS). This survey is an annual, cross-sectional survey of a probability sample drawn from Gfk’s KnowledgePanel, a probability-based web panel designed to be representative of non-institutionalized USA adults. Only adults sampled via address-based sampling or random digit dialing (previous) are eligible to join KnowledgePanel. Recruited panelists without internet access are provided a computer with internet access. Information from the profile survey is used to calculate a panel demographic post-stratification weight to adjust for sources of sampling and non-sampling error, such as panel recruitment non-response and panel attrition. Data collection occurred June–November, 2014. Participants completed the main survey in 23 min (median) and received a cash-equivalent of $5 for their participation. This study was approved by the Georgia State University Institutional Review Board.

### Sample

A probability sample of USA adults from KnowledgePanel and a representative oversample of pre-identified cigarette smokers were selected with probabilities proportional to size (PPS) after application of the panel demographic post-stratification weight. Overall, we invited 7991 KnowledgePanel members to participate in the survey: 7061 members for the general population sample, of which 74.3 % completed the screener survey and qualified for the main survey; and 930 members for the smoker augment sample, of which 697 completed the screener and 599 (74.9 %) qualified for the main survey by confirming their current smoking status. Of 5833 qualified completers, 116 cases were excluded due to refusing to answer more than one-half of the survey questions, yielding an analytic sample of 5717 cases. A final stage completion rate of 74.4 % and a qualification rate of 98.2 % were obtained. The average panel recruitment rate for this study, reported by GfK, was 13.7 % and the average profile rate was 65.3 %, for a cumulative response rate of 6.6 %. Following closure of the main survey field period, a study-specific post-stratification weight was computed using an iterative proportional fitting (raking) procedure to adjust for survey non-response as well as for oversampling of smokers. Demographic and geographic distributions from the most recent Current Population Survey (CPS) were employed as benchmarks for adjustment, and included gender, age, race/ethnicity, education, household income, census region, metropolitan area, and internet access.

### Measures

#### Smoking status

Respondents that reported not having smoked at least 100 cigarettes in their lives were classified as never (established) smokers. Those respondents who reported smoking at least 100 cigarettes in their lives were asked, “Do you currently smoke cigarettes every day, some days, or not at all?” They were classified as current smokers if they reported currently smoking cigarettes “every day” or “some days” and as former smokers if they reported “not at all”. Recent former smokers were defined as former smokers who reported the last time they smoked a cigarette, even one or two puffs, was within the past 5 years, and non-recent former smokers were defined as former smokers who last smoked a cigarette more than 5 years ago.

#### Awareness and use of ENDS and other tobacco products

Awareness and use of ENDS and other tobacco products (namely, chewing tobacco, snuff, or dip; large, premium cigars; LCCs; snus; dissolvables; and hookahs) were assessed by asking respondents if they had heard of the product before taking the survey and, if so, whether they had ever tried the product, even just one time. Those respondents who indicated they have tried one or more of the products were asked whether they had used the products at least once during the past 30 days. Current users of these products were defined as those who had used the product at least once during the past 30 days. For some analyses, ever (non-current) users were defined as those who reported ever use of the product but no use in the past 30 days.

Prior to the questions assessing awareness and use of these products, respondents were shown descriptions and images of ENDS and LCCs. The description for ENDS used “e-cigarette” and referred broadly to electronic nicotine delivery systems. The description provided for LCCs characterized them as smaller than traditional cigars, usually brown, and listed several common brands.

#### Respondent characteristics

Demographic and other respondent characteristics data were obtained from profile surveys administered by GfK to KnowledgePanel panelists. Respondent characteristic included sex, age, race/ethnicity, educational attainment, annual household income, USA Census region, perceived health status, sexual orientation, and presence of a child in the home.

### Statistical analysis

We used Stata/MP (v.13.1) to obtain design-based (weighted) point estimates and 95 % confidence intervals for awareness and use of ENDS, combustible, and non-combustible tobacco products. Associations among variables were tested using weighted logistic regression models and Rao–Scott *χ*^2^ tests (Rao and Scott 1981). Prior to conducting these analyses, we assessed the extent and ignorability of missing data for ever use and past 30-day use questions for the tobacco products. Pearson Chi-square tests of the missing completely at random (MCAR) (Fuchs 1982) assumption were conducted using Mplus (v.7.3) and were non-significant (ps > 0.99). As an additional check, full information maximum likelihood estimates of the weighted proportions of using each product under the missing at random (MAR) assumption were compared to the corresponding MCAR estimates. Differences in estimates were less than 0.5 %. On the bases of these checks, respondents with missing data were excluded from further analyses under the supported assumption that missingness is ignorable and completely at random. (See supplemental document for an expanded summary of the missing data.)

## Results

Table 1 reports awareness, ever use, and current use of ENDS and other novel and alternative tobacco products overall and by cigarette smoking status. Table 2 reports ever and current use of ENDS and current smoking by demography. Table 3 reports the adjusted associations between cigarette smoking status, other combustible tobacco use, and other non-combustible tobacco use with ENDS use. A detailed summary of the sample and reference population demographics and ENDS use by demography for emerging adults (18–24 years) and adults over 25 years old can be found in the supplemental document.

**Table 1:** Awareness and use of electronic nicotine delivery systems (ENDS) and other tobacco products by cigarette smoking status among USA adults, 2014

Tobacco products	*N*	Overall	Cigarette smoking status		
			Current smoker	Former smoker	Never smoker
		% (95 % CI)	% (95 % CI)	% (95 % CI)	% (95 % CI)
Electronic nicotine delivery systems					
Awareness	5629	91.9 (91.0, 92.7)	89.9 (87.5, 91.8)	91.9 (90.2, 93.4)	92.5 (91.3, 93.6)
Ever use**	5085	14.9 (13.9, 16.0)	51.1 (47.8, 54.4)	13.1 (11.3, 15.2)	4.7 (3.8, 5.8)
Past 30-day use**	5008	4.9 (4.3, 5.5)	20.7 (18.1, 23.6)	3.8 (2.8, 5.1)	0.9 (0.5, 1.5)
Combustible tobacco					
Little cigars/cigarillos					
Awareness**	5629	87.3 (86.2, 88.3)	88.7 (86.2, 90.7)	92.5 (90.8, 93.9)	84.2 (82.6, 85.8)
Ever use**	5111	30.6 (29.2, 32.0)	46.8 (43.5, 50.1)	48.5 (45.7, 51.2)	16.5 (15.0, 18.1)
Past 30-day use**	5090	2.8 (2.3, 3.4)	9.4 (7.5, 11.8)	2.1 (1.4, 3.1)	1.2 (0.8, 2.0)
Hookah					
Awareness	5629	81.4 (80.2, 82.5)	83.8 (81.2, 86.1)	80.8 (78.5, 82.9)	80.9 (79.2, 82.5)
Ever use**	5155	13.3 (12.3, 14.4)	21.0 (18.4, 23.8)	16.0 (14.1, 18.1)	9.6 (8.3,11.0)
Past 30-day use	5143	1.2 (0.9, 1.6)	2.0 (1.2, 3.4)	1.0 (0.5, 1.8)	1.0 (0.6, 1.7)
Large cigars					
Awareness*	5629	90.5 (89.5, 91.4)	88.3 (85.8, 90.3)	92.8 (91.1, 94.2)	90.0 (88.6, 91.3)
Ever use**	5120	26.0 (24.7, 27.3)	30.9 (28.0, 33.9)	41.2 (38.5, 43.9)	16.4 (15.0, 18.0)
Past 30-day use	5088	1.8 (1.4, 2.2)	2.7 (1.8, 3.9)	2.0 (1.3, 2.9)	1.4 (1.0, 2.0)
Non-combustible tobacco					
Smokeless tobacco					
Awareness**	5629	93.8 (92.9, 94.5)	90.7 (88.5, 92.3)	95.2 (93.7, 96.3)	94.0 (92.8, 95.0)
Ever use**	5078	17.9 (16.8, 19.0)	25.3 (22.6, 28.2)	27.4 (25.0, 29.9)	10.6 (9.4, 11.9)
Past 30-day use*	5054	2.1 (1.7, 2.6)	3.5 (2.4, 5.1)	2.4 (1.6, 3.6)	1.4 (1.0, 2.0)
Snus					
Awareness**	5629	59.7 (58.3, 61.1)	80.5 (77.9, 82.9)	58.5 (55.8, 61.1)	54.1 (52.1, 56.2)
Ever use**	5296	4.0 (3.5, 4.7)	11.1 (9.2, 13.4)	5.2 (4.1, 6.6)	1.4 (1.0, 2.0)
Past 30-day use**	5295	0.3 (0.2, 0.6)	1.2 (0.5, 2.8)	0.3 (0.1, 0.9)	0.0 (0, 0.2)
Dissolvables					
Awareness**	5629	40.6 (39.2, 42.0)	57.8 (54.7, 60.9)	39.3 (36.7, 42.0)	36.1 (34.2, 38.0)
Ever use**	5387	1.1 (0.8, 1.4)	3.3 (2.3, 4.6)	1.0 (0.6, 1.7)	0.5 (0.3, 1.0)
Past 30-day use		–	–	–	–

% = weighted overall and column percentages. There were not sufficient data to obtain reliable estimates of past 30-day use of dissolvables

*N* unweighted counts, *CI* confidence interval

* *p* < 0.01, ** *p* < 0.001 (Rao–Scott *χ* ^2^)

**Table 2:** Estimated percentage of use of electronic nicotine delivery systems (ENDS) and of cigarettes among USA adults, 2014

Demographic characteristics	Overall	ENDS		Cigarette-current use
	Total (*N* = 5717)	Ever use	Current use	
	% (95 % CI)	% (95 % CI)	% (95 % CI)	% (95 % CI)
Overall	–	14.9 (13.9, 16.0)	4.9 (4.3, 5.5)	16.6 (15.6, 17.6)
Sex		*p* = 0.22	*p* = 0.20	***p*** **=** **0.015**
Male	48.1 (46.7, 49.6)	15.6 (14.1, 17.2)	5.3 (4.4, 6.3)	17.8 (16.4, 19.4)
Female	51.9 (50.4, 53.3)	14.2 (12.9, 15.8)	4.5 (3.7, 5.4)	15.4 (14.1, 16.7)
Age (years)		***p*** **<** **0.001**	***p*** **<** **0.001**	***p*** **<** **0.001**
18–24	12.6 (11.5, 13.8)	19.9 (16.1, 24.2)	5.2 (3.4, 7.9)	13.6 (10.9, 16.9)
25–34	16.3 (15.2, 17.4)	23.3 (20.1, 26.7)	8.6 (6.6, 11.0)	20.7 (18.1, 23.7)
35–44	18.1 (16.9, 19.2)	16.2 (13.8, 18.9)	5.1 (3.8, 6.8)	18.9 (16.4, 21.7)
45–54	16.4 (15.4, 17.5)	15.1 (12.9, 17.6)	4.8 (3.6, 6.3)	21.4 (19.0, 24.0)
55–64	18.9 (17.9, 20.0)	11.3 (9.6, 13.2)	4.0 (3.0, 5.2)	16.3 (14.5, 18.4)
65+	17.7 (16.8, 18.8)	6.3 (5.0, 8.0)	2.2 (1.5, 3.2)	8.3 (6.9, 9.8)
Race/ethnicity		*p* = 0.62	*p* = 0.81	***p*** **<** **0.001**
White, NH	66.0 (64.5, 67.5)	15.0 (13.9, 16.3)	4.8 (4.1, 5.5)	15.2 (14.2, 16.3)
Black, NH	11.6 (10.6, 12.6)	12.8 (10.2, 16.0)	4.5 (3.0, 6.6)	26.7 (23.1, 30.7)
Hispanic	15.0 (13.8, 16.2)	15.3 (12.2, 19.0)	5.2 (3.5, 7.8)	15.9 (13.2, 19.1)
Other, NH	7.5 (6.7, 8.4)	16.1 (11.8, 21.5)	5.9 (3.6, 9.6)	14.1 (10.9, 18.1)
Education		***p*** **<** **0.001**	***p*** **=** **0.003**	***p*** **<** **0.001**
<High school	12.6 (11.4, 13.8)	19.5 (15.8, 23.7)	6.4 (4.5, 9.1)	28.8 (24.7, 33.3)
High school	29.6 (28.3, 30.9)	15.2 (13.4, 17.3)	5.1 (4.1, 6.4)	20.2 (18.3, 22.2)
Some college	28.9 (27.7, 30.2)	19.2 (17.2, 21.4)	5.8 (4.7, 7.1)	18.3 (16.6, 20.1)
College degree+	28.9 (27.7, 30.2)	8.4 (7.1, 9.8)	3.1 (2.4, 4.1)	5.8 (4.9, 6.8)
Household income		***p*** **<** **0.001**	***p*** **<** **0.001**	***p*** **<** **0.001**
<$15 K	11.4 (10.4, 12.4)	23.4 (19.6, 27.6)	8.2 (6.0, 11.2)	37.0 (32.8, 41.4)
$15 K–$24.9 K	7.1 (6.4, 7.9)	16.3 (12.8, 20.4)	5.8 (3.8, 8.7)	24.5 (20.4, 29.2)
$25 K–$39.9 K	15.5 (14.4, 16.6)	15.7 (13.1, 18.6)	5.6 (4.2, 7.5)	21.4 (18.7, 34.4)
$40 K–$59.9 K	16.2 (15.2, 17.3)	15.5 (13.1, 18.2)	5.2 (3.8, 7.0)	17.4 (15.2, 19.8)
$60 K–$84.9 K	16.6 (15.6, 17.7)	14.7 (12.3, 17.5)	4.8 (3.5, 6.6)	12.2 (10.3, 14.3)
$85 K–$99.9 K	6.8 (6.1, 7.6)	18.4 (14.2, 23.5)	6.5 (4.1, 10.0)	11.7 (8.9, 15.3)
$100 K+	26.4 (25.1, 27.6)	9.3 (7.9, 11.1)	2.3 (1.7, 3.2)	6.3 (5.3, 7.5)
USA region		*p* = 0.56	*p* = 0.24	***p*** **=** **0.010**
Northeast	18.2 (17.1, 19.3)	15.0 (12.6, 17.8)	4.1 (2.9, 5.8)	17.0 (14.7, 19.5)
Midwest	21.4 (20.3, 22.5)	14.2 (12.4, 16.3)	4.5 (3.4, 5.9)	17.1 (15.3, 19.1)
South	37.1 (35.7, 38.5)	14.4 (12.8, 16.2)	4.8 (3.9, 5.8)	17.9 (16.3, 19.7)
West	23.4 (22.1, 24.7)	16.2 (14.0, 18.8)	6.0 (4.7, 7.7)	13.6 (11.8, 15.7)
Perceived health status		***p*** **<** **0.001**	***p*** **<** **0.001**	***p*** **<** **0.001**
Excellent	11.5 (10.6, 12.5)	9.7 (7.2, 13.0)	2.1 (1.2, 3.9)^†^	8.7 (6.6, 11.6)
Very good	36.3 (34.9, 37.7)	12.5 (11.0, 14.3)	4.1 (3.2, 5.1)	12.3 (10.9, 13.8)
Good	38.0 (36.6, 39.4)	15.9 (14.2, 17.7)	5.1 (4.2, 6.2)	20.2 (18.5, 22.1)
Fair	12.0 (11.0, 13.0)	21.5 (18.1, 25.4)	8.6 (6.4, 11.5)	25.6 (22.2, 29.3)
Poor	2.2 (1.8, 2.7)	26.9 (18.8, 36.9)	10.1 (5.5, 17.7)	39.4 (30.7, 48.9)
Sexual orientation		***p*** **<** **0.001**	*p* = 0.10	***p*** **=** **0.004**
Heterosexual	94.5 (93.7, 95.1)	14.4 (13.3, 15.5)	4.8 (4.2, 5.4)	18.6 (15.2, 17.2)
Gay/lesbian/bisexual/other	5.5 (4.9, 6.3)	24.1 (19.2, 29.8)	6.7 (4.5, 9.9)	22.8 (18.2, 28.0)
Presence of children under 18 in the household		*p* = 0.083	*p* = 0.092	*p* = 0.74
Yes	31.7 (30.3, 33.1)	16.3 (14.4, 18.5)	5.7 (4.5, 7.1)	16.8 (15.1, 18.7)
No	68.3 (66.9, 69.7)	14.3 (13.1, 15.5)	4.5 (3.9, 5.3)	16.5 (15.3, 17.7)

Current use of ENDS was defined as any use in the past 30 days. Boldface indicates statistical significance (*p* < 0.05)

*NH* non-Hispanic, *CI* confidence interval, *RSE* relative standard error

^†^30 % ≤ RSE ≤ 49 %

**Table 3:** Associations between combustible and other non-combustible tobacco use and use of electronic nicotine delivery systems (ENDS) among USA adults, 2014

Model predictors	ENDS ever use		ENDS current use	
	Model 1	Model 2	Model 1	Model 2
	AOR (95 % CI)	AOR (95 % CI)	AOR (95 % CI)	AOR (95 % CI)
Tobacco use				
Cigarette smoking status				
Current smokers	17.9*** (13.8, 23.3)	25.0*** (18.4, 34.0)	22.8*** (13.1, 39.9)	28.1*** (15.9, 49.4)
Former smokers	1.9*** (1.4, 2.6)	3.0*** (2.1, 4.2)	3.5*** (1.9, 6.5)	4.6*** (2.4, 8.6)
Never smokers	Ref	Ref	Ref	Ref
Other combustible product use				
Ever use	3.9*** (3.0, 5.0)	3.9*** (3.0, 5.1)	–	–
Never use	Ref	Ref	–	–
Current use	–	–	4.4*** (2.5, 7.7)	4.3*** (2.4, 7.9)
Ever (not current) use	–	–	1.8** (1.3, 2.6)	1.5* (1.0, 2.2)
Never use	–	–	Ref	Ref
Non-combustible product use				
Ever use	1.5** (1.2, 1.9)	1.6*** (1.2, 2.1)	–	–
Never use	Ref	Ref	–	–
Current use	–	–	1.9 (1.0, 3.7)	1.5 (0.7, 3.1)
Ever (non-current) use	–	–	1.1 (0.8, 1.6)	1.2 (0.8, 1.8)
Never use	–	–	Ref	Ref
Demography and health status covariates				
Sex				
Male	–	Ref	–	Ref
Female	–	1.9*** (1.5, 2.3)	–	1.3 (0.9, 1.8)
Age (years)				
18–24	–	Ref	–	Ref
25–34	–	0.7 (0.4, 1.1)	–	0.9 (0.5, 1.7)
35–44	–	0.4*** (0.2, 0.6)	–	0.5* (0.2, 0.9)
45–54	–	0.3*** (0.2, 0.4)	–	0.4** (0.2, 0.7)
55–64	–	0.2*** (0.1, 0.3)	–	0.4* (0.2, 0.8)
65+	–	0.1*** (0.1, 0.2)	–	0.3** (0.1, 0.6)
Race/ethnicity				
White, NH	–	Ref	–	Ref
Black, NH	–	0.5** (0.3, 0.8)	–	0.6 (0.3, 1.1)
Hispanic	–	1.0 (0.7, 1.5)	–	1.1 (0.6, 1.8)
Other, NH	–	1.0 (0.6, 1.6)	–	1.2 (0.7, 2.3)
Education				
<High school	–	Ref	–	Ref
High school	–	1.0 (0.7, 1.5)	–	1.2 (0.7, 2.1)
Some college	–	1.3 (0.9, 2.0)	–	1.4 (0.8, 2.4)
College degree +	–	0.8 (0.5, 1.2)	–	1.6 (0.9, 3.1)
Household income				
<$15 K	–	Ref	–	Ref
$15 K–$24.9 K	–	1.1 (0.6, 1.9)	–	1.3 (0.6, 2.6)
$25 K–$39.9 K	–	1.1 (0.7, 1.7)	–	1.3 (0.7, 2.1)
$40 K–$59.9 K	–	1.3 (0.9, 2.0)	–	1.3 (0.8, 2.3)
$60 K–$84.9 K	–	1.4 (0.9, 2.1)	–	1.3 (0.7, 2.5)
$85 K–$99.9 K	–	2.0** (1.2, 3.2)	–	2.1* (1.0, 4.5)
$100 K+	–	1.1 (0.7, 1.7)	–	0.9 (0.5, 1.7)
USA region				
Northeast	–	Ref	–	Ref
Midwest	–	0.8 (0.6, 1.1)	–	1.0 (0.6, 1.7)
South	–	0.8 (0.6, 1.1)	–	1.1 (0.7, 1.8)
West	–	1.1 (0.7, 1.5)	–	1.8* (1.1, 2.9)
Perceived health status				
Excellent	–	0.6 (0.3, 1.4)	–	0.3* (0.1, 0.9)
Very good	–	0.7 (0.3, 1.3)	–	0.6 (0.3, 1.2)
Good	–	0.7 (0.4, 1.4)	–	0.6 (0.3, 1.3)
Fair	–	1.1 (0.5, 2.1)	–	1.1 (0.5, 2.3)
Poor	–	Ref	–	Ref
Sexual orientation				
Gay/lesbian/bisexual/Other	–	1.4 (0.9, 2.1)	–	1.1 (0.6, 1.9)
Heterosexual	–	Ref	–	Ref
Presence of children under 18 in the household				
Yes	–	Ref	–	Ref
No	–	1.1 (0.8, 1.4)	–	0.8 (0.6, 1.2)

Model 1 included cigarette smoking status and other combustible and non-combustible tobacco use as predictors. Model 2 includes model 1 predictors, and demographic and health status covariates as statistical controls. Other combustible products include hookah, large cigars and little cigars and cigarillos. Non-combustible products include snus, dissolvables and smokeless tobacco products. Current use was defined as use in the previous 30 days, and ever (not current) use was defined as having ever used the product but not in the past 30 days

Boldface indicates statistical significance (*p* < 0.05)

*AOR* adjusted odds ratio, *CI* confidence interval

* *p* < 0.05, ** *p* < 0.01, *** *p* < 0.001

^†^30 % ≤ RSE ≤ 49 %

### Awareness and use of electronic nicotine delivery systems (ENDS)

Overall, 91.9 % of USA adults had heard of ENDS. Our study estimated 14.9 % of USA adults have ever used ENDS and 4.9 % were current users. Use was substantially higher among current cigarette smokers compared to former and never smokers. Among current cigarette smokers, the odds of ever (OR = 21.4, 95 % CI = 16.5, 27.8) and current use (OR = 29.7, 95 % CI = 17.3, 51.0) of ENDS were substantially higher than for never smokers. Of those who have ever tried ENDS, 57.7 % (95 % CI = 53.7, 61.5) were current cigarette smokers, 25.3 % (95 % CI = 22.0, 28.9) were former smokers, and 17.0 % (95 % CI = 14.0, 20.6) were never smokers.

Ever and current use of ENDS is highest among young adults, particularly 25–34 year olds; those with some college education; those with less than a high school educational attainment; those with only fair or poor perceived health; and those with a non-heterosexual orientation. Young adults (25–34 years old) were more likely (OR = 1.7, 95 % CI = 1.0, 2.9) and those with a college degree were less likely (OR = 0.5, 95 % CI = 0.3, 0.7) to be current ENDS users. Adults with incomes between $40,000 and $60,000, $60,000 and $85,000, and greater than $100,000 had lower odds of current ENDS use (ORs = 0.3–0.6). Current ENDS use was less likely among those reporting good or better perceived physical health (OR = 0.5, 95 % CI = 0.3, 0.6), although this association was non-significant when controlling for cigarette smoking (AOR = 0.72, 95 % CI = 0.49, 1.05). Sex, race/ethnicity, presence of children in the home, and USA Census region were not associated with ENDS use in bivariate analyses. Sexual orientation was not associated with current ENDS use, though an association was observed for ever use. Non-heterosexual adults had higher odds (OR = 1.9, 95 % CI = 1.4, 2.6) of ever using ENDS. This association remained significant when controlling for smoking status (AOR = 1.8, 95 % CI = 1.2, 2.6).

### Awareness and use of other tobacco products

In 2014, an estimated 16.6 % (95 % CI = 15.6, 17.6) of USA adults were current cigarette smokers and 27.6 % (95 % CI = 26.3, 28.9) were former cigarette smokers. We observed high awareness of other combustible tobacco products (Table 1), and nearly two-thirds (62.3 %, 95 % CI = 60.9, 63.8) of USA adults were ever users and 21.6 % (95 % CI = 20.4, 22.8) were current users of any combustible tobacco product. Use of these other combustible products varied by cigarette smoking status. Nearly one-third of all respondents reported ever using LCCs. Ever use of LCCs was more likely among current or former cigarette smokers, and current users were more likely to be current cigarette smokers. More than one in ten respondents has ever used hookahs, with use being higher among current and former cigarette smokers. Few adults reported current hookah use (1.2 %). Approximately one-quarter of respondents have ever smoked large cigars, with ever use being more common among former cigarette smokers. Current use of large cigars was reported by few adults (1.8 %). Whereas USA adults reported very high awareness of traditional smokeless tobacco (93.8 %), their awareness of snus and dissolvables was comparatively low (59.7 and 40.6 %, respectively). Nearly one-fifth of USA adults (17.9 %) have ever used traditional smokeless tobacco, and 2.1 % are current users. Snus use was less prevalent, with 4.0 % reporting ever use and only 0.3 % reporting current use. Dissolvables were the least prevalent non-combustible tobacco product examined in this study; only 1.1 % of USA adults reported ever using the product. Current use of dissolvables was too low to obtain reliable estimates with our sample. Non-combustible tobacco use was more prevalent among current and former cigarette smokers compared to never smokers. Current cigarette smokers (OR = 2.9, 95 % CI = 2.3, 3.5) and former smokers (OR = 3.2, 95 % CI = 2.7, 3.8) were more likely to ever use traditional smokeless tobacco than never smokers. Current smokeless tobacco use was greater among current cigarette smokers (OR = 2.5, 95 % CI = 1.5, 4.2) and former smokers (OR = 1.7, 95 % CI = 1.0, 2.9). Regarding snus, ever use was several times greater among current cigarette smokers (OR = 9.0, 95 % CI = 5.9, 13.8) and former smokers (OR = 3.9, 95 % CI = 2.5, 6.2) than never smokers. Similar associations with smoking status were observed for current use of snus use and ever use of dissolvables.

### Associations between ENDS use and other tobacco use

Adjusting for other tobacco use, current smokers had nearly 18 times greater odds of ever use of ENDS and nearly 23 times the odds of current use of ENDS than did never smokers (Table 3, Model 1). Former smokers had twice the odds of ever ENDS use and 3.5 times the odds of current ENDS use as never smokers. These associations held and, in fact, were stronger, when statistically controlling for demographic and health status differences (Model 2). Other combustible and non-combustible use was also predictive of ENDS use. Ever use of any other combustible tobacco products (namely, LCCs, large cigars, and hookahs) was associated with 3.9 times greater odds of ever ENDS use than never use of these other combustible products. Ever users of non-combustible tobacco products (namely, smokeless tobacco, snus, and dissolvables) had 1.5 times greater odds of ever ENDS use than never users of these non-combustible tobacco products. Other combustible tobacco use was also predictive of current ENDS use. Adjusting for smoking status and non-combustible tobacco use, current users of non-combustible tobacco products had 4.4 times the odds and former use was associated with 1.8 times greater odds of current ENDS use than never users of these alternative combustible products. There was no statistically significant association between non-combustible tobacco product use and current ENDs use. These patterns of findings also remained and effect sizes undiminished after statistical adjustments were made for the demographic and health status covariates. After adjusting for other tobacco use and other covariates, sex, age, race/ethnicity, income, USA region, and perceived health status were significantly associated with ever use of ENDS and/or with current use of ENDS.

**Fig. 1:**
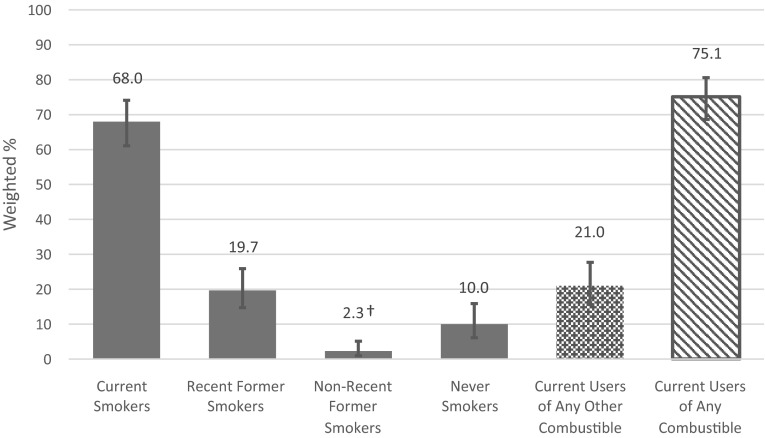
Tobacco product use among current users of electronic nicotine delivery systems in the USA, 2014 (*N* = 316). Former cigarette smokers were classified as recent former smokers if they report last cigarette use within the past 5 years and as non-recent former smokers if they reported last cigarette use more than 5 years ago. The *bars* *shaded in gray* represent mutually exclusive and exhaustive groups and sum to 100 % of current ENDS users. *Current users of any other combustible* include those who reported past 30-day use of little cigars or cigarillos, large cigars, or hookah. *Current users of any combustible* include those who reported any combustible tobacco use in the past 30 days or current cigarette smoking someday or everyday. *Error bars* depict 95 % confidence intervals. *RSE* relative standard error. ^†^30 % ≤ RSE ≤ 49 %

The findings from the logistic regression models are suggestive of high rates of dual use of ENDS and conventional cigarettes. Of those who were current ENDS users, 68.0 % were current cigarette smokers, 19.7 % were recent former smokers (quit within past 5 years), 2.3 % were non-recent former smokers (quit more than 5 years ago), and 10.0 % were never cigarette smokers (Fig. 1). Among current ENDS users, 21.0 % were current users of any alternative combustible tobacco product (namely, LCCs, large cigars, and/or hookah) and 75.1 % were current users of any combustible tobacco product (namely, LCCs, large cigars, hookah, and/or cigarettes).

## Discussion

The findings of this study provide current data about the awareness and use of ENDS and other novel tobacco products. Of particular interest, an estimated 1 in 20 USA adults, including 1 in 4 current cigarette smokers and 1 in 25 former smokers, were current ENDS users. These estimates are more than two times higher than 2012/2013 national estimates reported in a recently published study (King et al. 2015), although slightly lower than 2013 estimates from another study (McMillen et al. 2014). Of the alternative tobacco products assessed, ENDS had the highest prevalence of current use.

To a large degree, the observed patterns are being driven by market forces rather than by public health. The tobacco industry and the financial markets are noting that the tobacco product markets are entering a period of potentially dramatic product innovation and transformation (Herzog et al. 2015; Philip Morris International 2015). Published commentaries and editorials suggest that if public policy encourages these changes, the tobacco epidemic could be transformed and dramatically reduced (Abrams 2014; Cobb and Abrams 2014; Fagerstrom and Bridgman 2014; West and Brown 2014). The 2014 Surgeon General report acknowledged that additional “endgame strategies” could be needed to avert the projected and sustained pattern of smoking-attributable disease and premature death, but also noted, based upon the history of past tobacco industry driven product transformations, how the availability and promotion of these new nicotine delivery products could be projected either to reduce or increase population harm (US Department of Health and Human Services 2014). This potential impact that these products could have on population health was recently estimated in a dynamic population model (Vugrin et al. 2015). Recognizing that there was significant uncertainty in defining and selecting values for input parameters, the results showed how sensitive the model results were to (1) tobacco-related health risks of the various new and traditional products, (2) the rates of initiation of traditional and new products, and (3) the rates of switching and complete cessation, versus (4) sustained dual use of the novel products along with traditional cigarettes (Vugrin et al. 2015).

The results from this 2014 Tobacco Products and Risk Perceptions Survey and future annual surveys will provide nationally representative parameter estimates to inform such modeling of population harms. Specifically, the current data provide details about the manner in which ENDS are being combined (i.e., “dual use”) not only with cigarettes but with other combusted tobacco products. These high rates of dual use merit close monitoring to see if they could evolve into cessation of combusted tobacco product use or sustained dual use that delays individuals who would have quit tobacco products completely from actually quitting (Chen and Husten 2014). Second, the fact that almost one-third of the current ENDS users were ex-smokers and never smokers also merits careful monitoring. Of the 22.4 % of current ENDS users who were former cigarette smokers, about one in ten were long-term (more than 5 years) ex-smokers. This pattern raises concerns that the current marketing and promotion of ENDS are contributing to increased nicotine use and renormalization of tobacco use. Future surveys need to monitor these patterns to see if ex-smokers using ENDS are using them to prevent relapse or if their re-initiation of nicotine use could lead to relapsing back to combusted tobacco (McMillen et al. 2014).

This survey also shows that little cigars and cigarillos (LCC) had the highest overall prevalence of ever use among the alternative tobacco products, although this ranking of prevalence did not hold across smoking status subpopulations. Among smokeless products, chewing tobacco/snuff/dip tobacco was most common and dissolvables the least common in ever and current use. While the risk of tobacco-related diseases is known to vary across these products (US Department of Health and Human Services 2014), the risks of cigars, particularly the little cigars/cigarillos that are used and inhaled like cigarettes may have risks as high or exceeding the risks from smoking cigarettes (Chang et al. 2015). Thus, the data on the patterns of the use of these individual novel or alternative tobacco products or their use in combination with other combustible tobacco products can guide what additional evaluations of tobacco-related risk and risk perceptions are most needed.

### Limitations

This study has several limitations. First, the use of the internet panel may raise concerns about sample representativeness, especially if the panel has been used in prior tobacco research. Second, the data are based upon self-report, and biochemical verification of cigarette smoking and use of other products could not be conducted. While the validity of self-reported cigarette smoking has been confirmed (Caraballo et al. 2004; US Department of Health and Human Services 2014), the accuracy of self-report of other products, particularly the novel products, has not been evaluated and remains uncertain. Third, due to the rapidly changing nature of ENDS products being marketed and used, how accurately the questionnaire descriptions and terminology are assessing actual patterns of use is continuing to be evaluated.

### Conclusions

The results from this survey in conjunctions with other published data highlight that the patterns of trial and use of ENDS are evolving rapidly and merit continued surveillance and study. Use of ENDS is more common among current and former cigarette smokers, with patterns of use of little cigars/cigarillos, hookah, large cigars, smokeless tobacco, and ENDS varying significantly by sex, age, race/ethnicity, education, income, perceived health status, and sexual orientation. While use of ENDS primarily is combined with cigarette smoking, more than one-tenth of ENDS usage is among never cigarette smokers. Additionally, the patterns of higher rates of dual use of ENDS and combustible tobacco products among the less educated, lower income, and those with poorer perceived health are of concern. If these rates of dual use in these vulnerable populations do not contribute to increased smoking cessation then, when combined with the continuing high rates of use of all combusted tobacco products, these data raise significant public health concerns that the growing prevalence of ENDS use could contribute to increased population harm. Thus, this survey provides the FDA with timely population-level data that may help inform FDA policy development. Once FDA authority is properly extended, this survey provides the agency with evidence to inform product standards for novel nicotine products appropriate for the protection of public health, a standard specifically focused on evaluating product risk and benefit at a population level (Chen and Husten 2014; Husten and Deyton 2013).

## Electronic supplementary material

Below is the link to the electronic supplementary material.

Supplementary material 1 (DOCX 34 kb)
