# Supplementary material for: Use of electronic nicotine delivery systems and other tobacco products among USA adults, 2014: results from a national survey
Source: Int J Public Health. 2015 Nov 12;61:177–88. doi: 10.1007/s00038-015-0761-0 (PMC4819498; doi:10.1007/s00038-015-0761-0)
Supplement: Supplementary file 1 — Supplementary material 1 (DOCX 34 kb) [file 38_2015_761_MOESM1_ESM.docx]

**Supplemental Material:**

**Use of Electronic Nicotine Delivery Systems and Other Tobacco Products among USA Adults, 2014: Results from a National Survey**

Scott R. Weaver^1,2^

Ban A. Majeed^2^

Terry F. Pechacek^2,3^

Amy L. Nyman^2^

Kyle R. Gregory^2^

Michael P. Eriksen^2,3^

^1^Division of Epidemiology & Biostatistics, School of Public Health, Georgia State University, Atlanta, Georgia, USA

^2^Tobacco Center of Regulatory Science (TCORS), School of Public Health, Georgia State University, Atlanta, Georgia, USA

^3^Division of Health Management & Policy, School of Public Health, Georgia State University, Atlanta, Georgia, USA

**Address correspondence to:**

Scott R. Weaver, Ph.D.

Email: [srweaver@gsu.edu](mailto:srweaver@gsu.edu)

This supplemental document contains an expanded summary of the missing data, the demographic distributions of the sample and reference population (Table S1), and ENDS use by demography for emerging adults (18-24 years) and adults over 25 years old (Table S2).

**Method**

Statistical analysis

We used Stata/MP (v. 13.1; StataCorp LP, College Station, TX) to obtain design-based (weighted) point and 95% confidence intervals for awareness and use of ENDS, combustible, and non-combustible tobacco products. Bivariate associations among variables were tested using weighted logistic regression models and Rao-Scott χ^2^ tests (Rao and Scott 1981). Analyses were conducted in 2015.

Prior to conducting these analyses, we assessed the extent and ignorability of missing data for ever use and past 30 day use questions for tobacco products. The extent of missing data for these variables was greater than for other study variables due to a layout and programming of the ever use questions that resulted in some participants unintentionally skipping the question. These participants were invited to participate in a re-administration of the missed items. Additionally, a smaller subset of participants (unweighted *n* ≤ 93) with conflicting responses (e.g., reporting having used the product within the past 30 days while subsequently reporting a frequency of use of 0 days within the past 30 days) were excluded from analyses. After re-administration, the number of participants with missing data on the ever use measures for these products ranged from 330 (5.8%) for dissolvables to 632 (11.1%) for ENDS, and missingness on past 30 day use measures ranged from 320 (5.6%) for dissolvables to 709 (12.4%) for ENDS. Pearson chi-square tests of the missing completely at random (MCAR) (Fuchs 1982) assumption were conducted using Mplus (v. 7.3) and were non-significant (*ps* > .99). As an additional check, full information maximum likelihood estimates of the weighted proportions of using each product under the missing at random (MAR) assumption were compared to the corresponding MCAR estimates. Differences in estimates were less than 0.5%. On the bases of these checks, respondents with missing data were excluded from further analyses under the supported assumption that missingness is ignorable and completely at random (Little and Rubin 2014).

**References**

Fuchs C (1982) Maximum likelihood estimation and model selection in contingency tables with missing data. Journal of the American Statistical Association 77:270-278.

Little RJ, Rubin DB (2014) Statistical analysis with missing data. John Wiley & Sons,

Rao JN, Scott AJ (1981) The analysis of categorical data from complex sample surveys: chi-squared tests for goodness of fit and independence in two-way tables. Journal of the American Statistical Association 76:221-230.

**Table S1** Demographic characteristics of the survey participants, 2014 (USA Adults)

| Demographic characteristics | Unweighted counts | Population weighted counts | Weighted % (95% CI) |
| --- | --- | --- | --- |
| **Total** | 5,717 | — | — |
| Sex |  |  |  |
| Male | 2,807 | 113,572,773 | 48.1 (46.7, 49.6) |
| Female | 2,910 | 122,325,935 | 51.9 (50.4, 53.3) |
| Age (years) |  |  |  |
| 18-24 | 476 | 29,740,679 | 12.6 (11.5, 13.8) |
| 25-34 | 816 | 38,443,187 | 16.3 (15.2, 17.4) |
| 35-44 | 940 | 42,580,201 | 18.1 (16.9, 19.2) |
| 45-54 | 1,054 | 38,725,148 | 16.4 (15.4, 17.5) |
| 55-64 | 1,250 | 44,558,121 | 18.9 (17.9, 20.0) |
| 65+ |  | 41,851,372 | 17.7 (16.8, 18.8) |
| Race/Ethnicity |  |  |  |
| White, NH | 4,221 | 155,696,604 | 66.0 (64.5, 67.5) |
| Black, NH | 566 | 27,279,627 | 11.6 (10.6, 12.6) |
| Hispanic | 508 | 35,260,501 | 15.0 (13.8, 16.2) |
| Other, NH | 422 | 17,661,977 | 7.5 (6.7, 8.4) |
| Education |  |  |  |
| < High school | 442 | 29,671,933 | 12.6 (11.4, 13.8) |
| High school | 1,690 | 69,769,173 | 29.6 (28.3, 30.9) |
| Some college | 1,735 | 68,240,363 | 28.9 (27.7, 30.2) |
| College degree + | 1,850 | 68,217,239 | 28.9 (27.7, 30.2) |
| Household income |  |  |  |
| <$15K | 609 | 26,839,296 | 11.4 (10.4, 12.4) |
| $15K – $24.9K | 453 | 16,795,778 | 7.1 (6.4, 7.9) |
| $25K – $39.9K | 855 | 36,457,816 | 15.5 (14.4, 16.6) |
| $40K – $59.9K | 976 | 38,285,986 | 16.2 (15.2, 17.3) |
| $60K – $84.9K | 963 | 39,294,928 | 16.6 (15.6, 17.7) |
| $85K – $99.9K | 365 | 16,047,427 | 6.8 (6.1, 7.6) |
| $100K+ | 1,496 | 62,177,476 | 26.4 (25.1, 27.6) |
| USA region |  |  |  |
| Northeast | 1,019 | 42,890,511 | 18.2 (17.1, 19.3) |
| Midwest | 1,383 | 50,421,289 | 21.4 (20.3, 22.5) |
| South | 2,067 | 87,492,481 | 37.1 (35.7, 38.5) |
| West | 1,248 | 55,094,427 | 23.4 (22.1, 24.7) |
| Perceived health status |  |  |  |
| Excellent | 564 | 25,549,832 | 11.5 (10.6, 12.5) |
| Very good | 1,984 | 80,405,597 | 36.3 (34.9, 37.7) |
| Good | 2,091 | 84,201,101 | 38.0 (36.6, 39.4) |
| Fair | 645 | 26,533,688 | 12.0 (11.0, 13.0) |
| Poor | 131 | 4,920,423 | 2.2 (1.8, 2.7) |
| Sexual orientation |  |  |  |
| Heterosexual | 5,347 | 219,982,885 | 94.5 (93.7, 95.1) |
| Gay/Lesbian/ Bisexual/Other | 305 | 12,877,220 | 5.5 (4.9, 6.3) |
| Presence of children under 18 in the household |  |  |  |
| Yes | 1,647 | 74,709,985 | 31.7 (30.3, 33.1) |
| No | 4,070 | 161,188,723 | 68.3 (66.9, 69.7) |

**Table S2** Use of Electronic Nicotine Delivery Systems (ENDS) by Demography among Emerging Adults (18-24 Years) and Adults 25 Years or Older, 2014 (USA)

|  | 18-24 years old | |  | 25 years and older | |
| --- | --- | --- | --- | --- | --- |
| Characteristics | Ever use | Current use |  | Ever use | Current use |
|  | % (95% CI) | % (95% CI) |  | % (95% CI) | % (95% CI) |
| Overall | 19.9 (16.1, 24.2) | 5.2 (3.4, 7.9) |  | 14.2 (13.2, 15.3) | 4.8 (4.2, 5.5) |
| Sex | p=0.11 | p=0.35 |  | p=0.67 | p=0.33 |
| Male | 22.8 (17.5, 29.2) | 6.2 (3.6, 10.5) |  | 14.4 (13.0, 16.0) | 5.1 (4.3, 6.2) |
| Female | 16.3 (11.6, 22.4) | 4.1 (2.1, 8.0)† |  | 14 (12.6, 15.6) | 4.5 (3.7, 5.4) |
| Race | p=0.05 | p=0.11 |  | p=0.73 | p=0.82 |
| White, NH | 22.0 (17.3, 27.6) | 5.7 (3.4, 9.3) |  | 14.2 (13.0, 15.4) | 4.6 (4.0, 5.4) |
| Black, NH | 10.9 (4.2, 25.6)† | 1.1 (0.2, 7.6)‡ |  | 13.1 (10.3, 16.4) | 4.9 (3.2, 7.2) |
| Hispanic | 13.3 (7.1, 23.5)† | 3.4 (1.2, 10.1)‡ |  | 15.7 (12.3, 19.9) | 5.7 (3.7, 8.6) |
| Other, NH | 34.6 (18.5, 55.1) | 13.1 (4.2, 33.9)‡ |  | 13.5 (9.5, 18.8) | 4.9 (2.8, 8.3) |
| Education | p=0.25 | p=0.93 |  | **p<0.001** | **p<0.001** |
| <HS | 17.8 (11.0, 27.4) | 4.2 (1.6, 11.0) |  | 20.1 (16.0, 24.9) | 7.3 (5.0, 10.5) |
| HS | 21.4 (14.6, 30.3) | 5.5 (2.5, 11.8)† |  | 14.4 (12.6, 16.5) | 5.1 (4.0, 6.4) |
| Some college | 23.4 (17.4, 30.8) | 6.0 (3.2, 11.1)† |  | 18.5 (16.5, 20.8) | 5.7 (4.6, 7.1) |
| College + | 9.0 (3.2, 22.8)‡ | 4.6 (1.1, 17.5)‡ |  | 8.3 (7.0, 9.8) | 3.0 (2.3, 4.0) |
| Household income | p=0.34 | p=0.21 |  | **p<0.001** | **p<0.001** |
| <15K | 26.5 (16.6, 39.4) | 5.8 (1.8, 16.9)‡ |  | 22.8 (18.8, 27.3) | 8.6 (6.2, 11.9) |
| 15K-99.99K | 19.7 (14.7, 25.7) | 6.7 (4.0, 10.9) |  | 15.3 (14.0, 16.7) | 5.2 (4.5, 6.1) |
| 100K + | 17.0 (11.3, 24.8) | 2.4 (0.9, 6.3)‡ |  | 8.1 (6.7, 9.8) | 2.3 (1.6, 3.2) |
| USA region | p=0.36 | p=0.52 |  | p=0.43 | p=0.19 |
| Northeast | 27.3 (17.9, 39.4) | 7.5 (2.8, 18.2) |  | 13.3 (11.0, 16.1) | 3.7 (2.5, 5.3) |
| Midwest | 16.5 (10.4, 25.0) | 2.9 (0.9, 8.7) |  | 14.0 (12.1, 16.1) | 4.7 (3.6, 6.2) |
| South | 19.1 (13.3, 26.6) | 4.4 (2.2, 8.6)† |  | 13.7 (12.1, 15.5) | 4.8 (3.9, 5.9) |
| West | 18.4 (11.6, 28.0) | 6.8 (3.2, 13.9)† |  | 15.9 (13.6, 18.5) | 5.9 (4.5, 7.6) |
| Physical Health | p=0.49 | p=0.52 |  | **p<0.001** | **p<0.001** |
| Excellent/Very good | 19.1 (14.4, 24.9) | 4.9 (2.7, 8.6) |  | 10.6 (9.3, 12.1) | 3.4 (2.7, 4.3) |
| Good/Fair/Poor | 22.1 (16.0, 29.8) | 6.5 (3.4, 12.0)† |  | 17.2 (15.7, 18.9) | 6.1 (5.2, 7.2) |
| Sexual orientation | p=0.49 | p=0.34 |  | **p<0.001** | **p=0.024** |
| Not heterosexual | 24.4 (13.2, 40.6) | 2.5 (0.5, 11.4)‡ |  | 24.0 (18.9, 30.1) | 7.7 (5.1, 11.4) |
| Heterosexual | 19.6 (15.7, 24.3) | 5.4 (3.4, 8.3) |  | 13.7 (12.7, 14.8) | 4.7 (4.1, 5.4) |
| Presence of children under 18 in the household | p=0.40 | p=0.58 |  | p=0.21 | **p=0.03** |
| Yes | 22.0 (15.9, 29.7) | 4.5 (2.1, 9.3)† |  | 15.2 (13.3, 17.4) | 5.9 (4.7, 7.4) |
| No | 18.4 (14.0, 23.8) | 5.8 (3.4, 9.5) |  | 13.8 (12.6, 15.0) | 4.4 (3.7, 5.1) |
| Smoking status | **p<0.001** | **p<0.001** |  | **p<0.001** | **p<0.001** |
| Current smoker | 55.4 (42.9, 67.2) | 22.2 (12.9, 35.5) |  | 50.6 (47.2, 54.0) | 20.6 (17.9, 23.5) |
| Former smoker | 47.0 (28.7, 66.1) | 16.2 (6.0, 36.8)‡ |  | 12.1 (10.3, 14.1) | 3.4 (2.5, 4.6) |
| Never smoker | 10.9 (7.7, 15.2) | 1.8 (0.8, 4.1)‡ |  | 3.3 (2.5, 4.3) | 0.7 (0.4, 1.3)† |

Current use of ENDS was defined as use in past 30 days. Boldface indicates statistical significance (*p*<0.05). NH= non-Hispanic. CI = Confidence Interval. HS = High school. RSE = Relative Standard Error.

† 30% < RSE < 49%. ‡ RSE >50%
